# Directly converted patient-specific induced neurons mirror the neuropathology of FUS with disrupted nuclear localization in amyotrophic lateral sclerosis

**DOI:** 10.1186/s13024-016-0075-6

**Published:** 2016-01-22

**Authors:** Su Min Lim, Won Jun Choi, Ki-Wook Oh, Yuanchao Xue, Ji Young Choi, Sung Hoon Kim, Minyeop Nahm, Young-Eun Kim, Jinhyuk Lee, Min-Young Noh, Seungbok Lee, Sejin Hwang, Chang-Seok Ki, Xiang-Dong Fu, Seung Hyun Kim

**Affiliations:** Department of Translational Medicine, Graduate School of Biomedical Science and Engineering, Hanyang University, Seoul, 133-792 Republic of Korea; Cell Therapy Center, Hanyang University Hospital, Seoul, 133-792 Republic of Korea; Department of Neurology, Sheikh Khalifa Specialty Hospital, Ras Al Khaimah, United Arab Emirates; Department of Neurology, College of Medicine, Hanyang University, Seoul, 133-792 Republic of Korea; Key Laboratory of RNA Biology, Institute of Biophysics, Chinese Academy of Sciences, Beijing, 100101 China; Department of Laboratory Medicine and Genetics, Samsung Medical Center, Sungkyunkwan University School of Medicine, Seoul, 135-710 Republic of Korea; Korean Bioinformation Center, Korea Research Institute of Bioscience and Biotechnology, Daejeon, 305-806 Republic of Korea; Department of Bioinformatics, University of Sciences and Technology, Daejeon, 305-806 Republic of Korea; Department of Brain and Cognitive Sciences, College of Natural Sciences, Seoul National University, Seoul, 110-744 Republic of Korea; Department of Anatomy and Cell Biology, College of Medicine, Hanyang University, Seoul, 133-792 Republic of Korea; Department of Cellular Molecular Medicine, University of California, La Jolla, San Diego, CA 92093 USA

**Keywords:** Amyotrophic lateral sclerosis, Fused in sarcoma, Human cell models, Induced neuron, Nuclear localization signal, Stress granules, Neuronal cytoplasmic inclusion

## Abstract

**Background:**

Mutations in the fused in sarcoma (*FUS*) gene have been linked to amyotrophic lateral sclerosis (ALS). ALS patients with *FUS* mutations exhibit neuronal cytoplasmic mislocalization of the mutant FUS protein. ALS patients’ fibroblasts or induced pluripotent stem cell (iPSC)-derived neurons have been developed as models for understanding ALS-associated FUS (ALS-FUS) pathology; however, pathological neuronal signatures are not sufficiently present in the fibroblasts of patients, whereas the generation of iPSC-derived neurons from ALS patients requires relatively intricate procedures.

**Results:**

Here, we report the generation of disease-specific induced neurons (iNeurons) from the fibroblasts of patients who carry three different *FUS* mutations that were recently identified by direct sequencing and multi-gene panel analysis. The mutations are located at the C-terminal nuclear localization signal (NLS) region of the protein (p.G504Wfs*12, p.R495*, p.Q519E): two *de novo* mutations in sporadic ALS and one in familial ALS case. Aberrant cytoplasmic mislocalization with nuclear clearance was detected in all patient-derived iNeurons, and oxidative stress further induced the accumulation of cytoplasmic FUS in cytoplasmic granules, thereby recapitulating neuronal pathological features identified in mutant FUS (p.G504Wfs*12)-autopsied ALS patient. Importantly, such FUS pathological hallmarks of the patient with the p.Q519E mutation were only detected in patient-derived iNeurons, which contrasts to predominant FUS (p.Q519E) in the nucleus of both the transfected cells and patient-derived fibroblasts.

**Conclusions:**

Thus, iNeurons may provide a more reliable model for investigating FUS mutations with disrupted NLS for understanding FUS-associated proteinopathies in ALS.

**Electronic supplementary material:**

The online version of this article (doi:10.1186/s13024-016-0075-6) contains supplementary material, which is available to authorized users.

## Background

Fused in sarcoma (FUS) is a multifunctional DNA/RNA-binding protein involved in various aspects of cellular RNA metabolism and executes its main functions predominantly in the cell nucleus. Initially discovered as a fusion oncogene, mutations in the *FUS* gene resulting in FUS proteinopathies were recently linked to amyotrophic lateral sclerosis (ALS), responsible for ~4 % of familial and ~1 % of sporadic ALS cases [1–3]. *FUS* mutations cluster either in the glycine-rich region of the protein or in the RGG-rich C-terminal domain, where they disrupt the nuclear localization signal (NLS) and result in altered subcellular localization of the FUS protein. ALS-associated *FUS* (ALS-FUS) mutations have been reported to cause cytoplasmic mislocalization of the protein in the brain and spinal cord of ALS patients [4, 5]. Moreover, cytoplasmic FUS tends to aggregate to form inclusions in degenerating motor neurons of ALS patients [6–8]. As a consequence, both toxic gain-of-function in the cytoplasm and loss-of-function in the nucleus are proposed to be causative events in ALS development [9, 10].

Key pathological features have been documented based on immunocytochemical studies on cultured fibroblasts from ALS patients or immunohistological analysis on autopsy samples [11, 12]. These studies revealed abnormal cytoplasmic mislocalization of the FUS protein in ALS patients with *FUS* mutations in its NLS. When modeled on fibroblasts, however, mutant FUS proteins were predominantly detected in the nucleus, with minimal association with pathological signatures detected with those mutations in vivo [11, 13, 14]. Patient-derived induced pluripotent stem cells (iPSC) with the ability to differentiate into neural cells were found to be suitable for studying ALS-FUS pathology [15], but neuronal induction and differentiation processes using iPSC require tedious and labor-intensive procedures. Hence, it would be advantageous to develop rapid and simple FUS-associated ALS patient-derived cell models to study ALS-related neuronal pathology.

To overcome the limitations associated with the current cell modeling systems, we examined FUS pathology in a more disease-relevant cell model. We used our previously described method of repressing a polypyrimidine-tract-binding (PTB) protein to directly convert patient fibroblasts carrying *FUS* mutations and those from age-matched healthy controls into functional neurons (iNeuron) [16]. We have recently identified *FUS* mutations (p.G504Wfs*12, p.R495*, and p.Q519E) by direct sequencing and multi-gene panel testing [17–19]. In this study, we examined the pathophysiological and biochemical properties of the three different *FUS* mutations at NLS region. Analysis of brain and spinal cord autopsy samples from FUS (p.G504Wfs*12) patient demonstrated the expected pathologic features including nuclear clearance and cytoplasmic accumulation of FUS in neurons. To generate a cell model that recapitulates key pathological features found in autopsy, we compared cellular localization and aggregation-prone properties of the endogenous FUS in fibroblasts, HEK-293 cells and rat primary cortical neurons and directly converted iNeurons in the presence or absence of stress. Directly converted iNeurons from patient fibroblasts was the only model that recapitulated the mutant FUS-associated neurological pathology that is observed in autopsied brain and spinal cord. Moreover, we showed that the FUS neuropathology of the familial ALS patient with p.Q519E mutation could be demonstrated in directly converted iNeurons but not in transfected cells or patient-derived fibroblasts. These findings suggest that directly converted iNeurons have a potential to become reliable disease-relevant models for dissecting pathophysiologies of FUS-related proteinopathies in ALS.

## Results and discussion

### Clinical and genetic characteristics of three ALS patients harboring *FUS* mutations in the NLS region

**Fig. 1:**
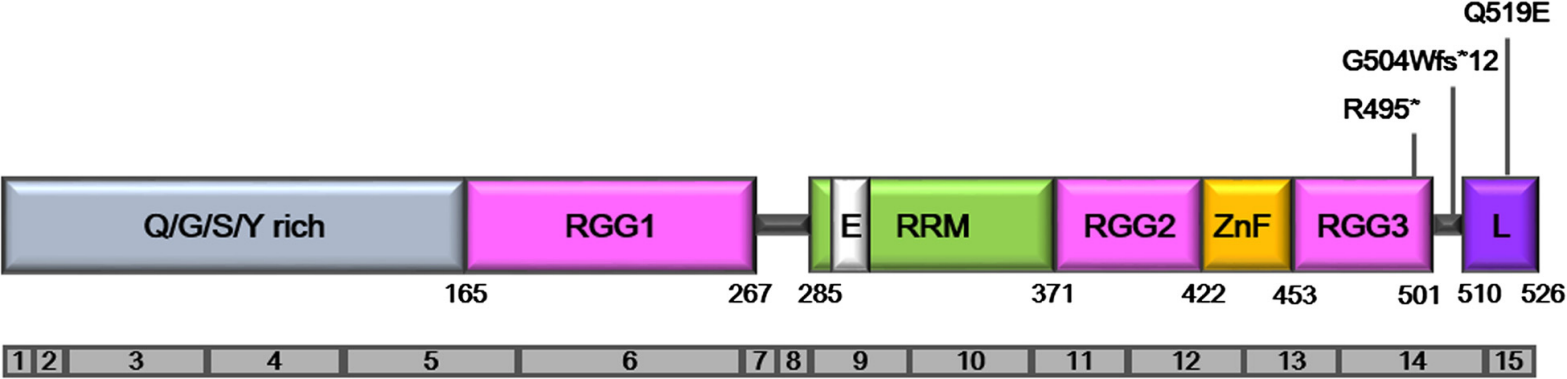
Schematic diagram of functional domains of the FUS protein with gene mutations identified in patients with ALS are provided. All three patients enrolled in this study have *FUS* mutations (p.G504Wfs*12, p.R495*, p.Q519E) that affect the NLS region. Q/G/S/Y rich = Gln/Gly/Ser/Tyr-rich domain; RGG = Arg/Gly/Gly-rich motifs; E = nuclear export signal; RRM = RNA-recognition motif; ZnF = zinc-finger motif; L = nuclear localization signal

Among ten diverse, recently identified FUS mutants or variants [17, 19], two *de novo* FUS mutants (p.G504Wfs*12, p.R495*) confirmed by trio study in sporadic ALS [18] and one FUS variant (p.Q519E) in familial ALS [19] were included in this study. The residues of the three mutants are located in the C-terminal region containing the nuclear localization signal (NLS). As diagrammed in Fig. 1, the Q519E mutation is a missense mutation in the C-terminal NLS region; the mutation (p.G504Wfs*12) causes a frame shift in exon 15, leading to a truncated FUS; and the mutation (p.R495*) creates a premature codon to eliminate the NLS. The mutation (p.R495*) is associated with an aggressive clinical phenotype of ALS [20–22], and the mutation (p.G504Wfs*12) is a pathogenic truncation mutant associated with sporadic ALS [18, 23]. In order to investigate whether FUS (p.Q519E) variant has the significance in disease pathogenesis, we established structural analysis of the mutation with Transportin-1 (Protein Data Bank, PDB (ID: 4FDD)) (Additional file 1: Figure S1). FUS is a nuclear protein that its nuclear import is mediated by interaction between Transportin-1 and the C-terminal NLS region of FUS [24, 25]. Hence, we analyzed the hydrogen bonding pattern of FUS-Transportin-1 complexes and observed that one hydrogen bond is relevant to the FUS Q519 residue. The distance between acceptor atom (oxygen; atom type: OE1) of E509 from Transportin-1 and donor atom (nitrogen; atom type: NE2) of Q519 from FUS was measured as 3.21 Å. Since the experimental structure has no hydrogen atoms, the angle of hydrogen bond was measured between acceptor (OE1 of E509 from Transportin-1), donor (NE2 of Q519 from FUS), and the prior atom connected on donor (CD of Q519 from FUS), which comes to 134.7°. This is a possible hydrogen bonding between FUS-Transportin-1 complexes. If the Q519 on FUS is mutated to E519, the length of side chain is decreased by one carbon chain (from 4 to 3) and the polar property changes to negative from neutral. In the end, the found Q519 (FUS)-E509 (Transportin-1) hydrogen bond in wild-type will disappear in the Q519E mutant. In addition, the negative-negative repulsion (E519-E509) in Q519E mutant may result in the deactivation on FUS-Transportin-1 binding, thus providing a significance of the FUS (p.Q519E) variant in disease pathogenesis.

Detailed clinical and epidemiological characteristics of three ALS patients with different *FUS* mutations, one sporadic ALS patient, and four healthy controls enrolled in this study are summarized in Table 1.

**Table 1:** Patients and controls whose skin fibroblasts were studied

Characteristic	ID	MND	Sex	Exon	*FUS* genotype	Age at biopsy, yr	Age of onset, yr	Familial history	Site of onset	ALSFRS-R	delta-FS	Survival, mo	Autopsy
ALS-FUS	HS374	777	M	15	Q519E^a^	34	34	Yes	Limb	46	0.10	>61 ^c^	N/A
	HS131	402	F	14	G504Wfs*12^b^	34	31	No	Limb	36	0.92	46	Yes
	HS197	502	F	14	R495*^b^	31	27	No	Bulbar	23	1.92	54	N/A
Sporadic ALS	HS250	551	M	N/A	N/A	N/A	57	No	N/A	39	0.82	68	Yes
CTL 1	N/A	N/A	F	N/A	N/A	35	N/A	N/A	N/A	N/A	N/A	N/A	N/A
CTL 2	N/A	N/A	F	N/A	N/A	40	N/A	N/A	N/A	N/A	N/A	N/A	N/A
CTL 3	N/A	N/A	M	N/A	N/A	45	N/A	N/A	N/A	N/A	N/A	N/A	N/A
CTL 4	N/A	N/A	N/A	N/A	N/A	N/A	N/A	N/A	N/A	N/A	N/A	N/A	Purchased

*FUS*, fused in sarcoma, *ALSFRS-R* a revised ALS functional rating scale, *ALS* amyotrophic lateral sclerosis, *F* female, *N/A* not applicable, *M* male, *CTL* control

ALSFRS-R and delta FS were evaluated at the first visit

^a^Reported previously (Kim H-J et al., 2015). ^b^Reported previously (Kwon MJ et al., 2012; Kim YE et al., 2014).

^c^>61 means that more than 61 months have passed since symptom onset and the patient is still alive on the last follow-up

### FUS pathology in ALS-FUS patient brain and spinal motor neurons

**Fig. 2:**
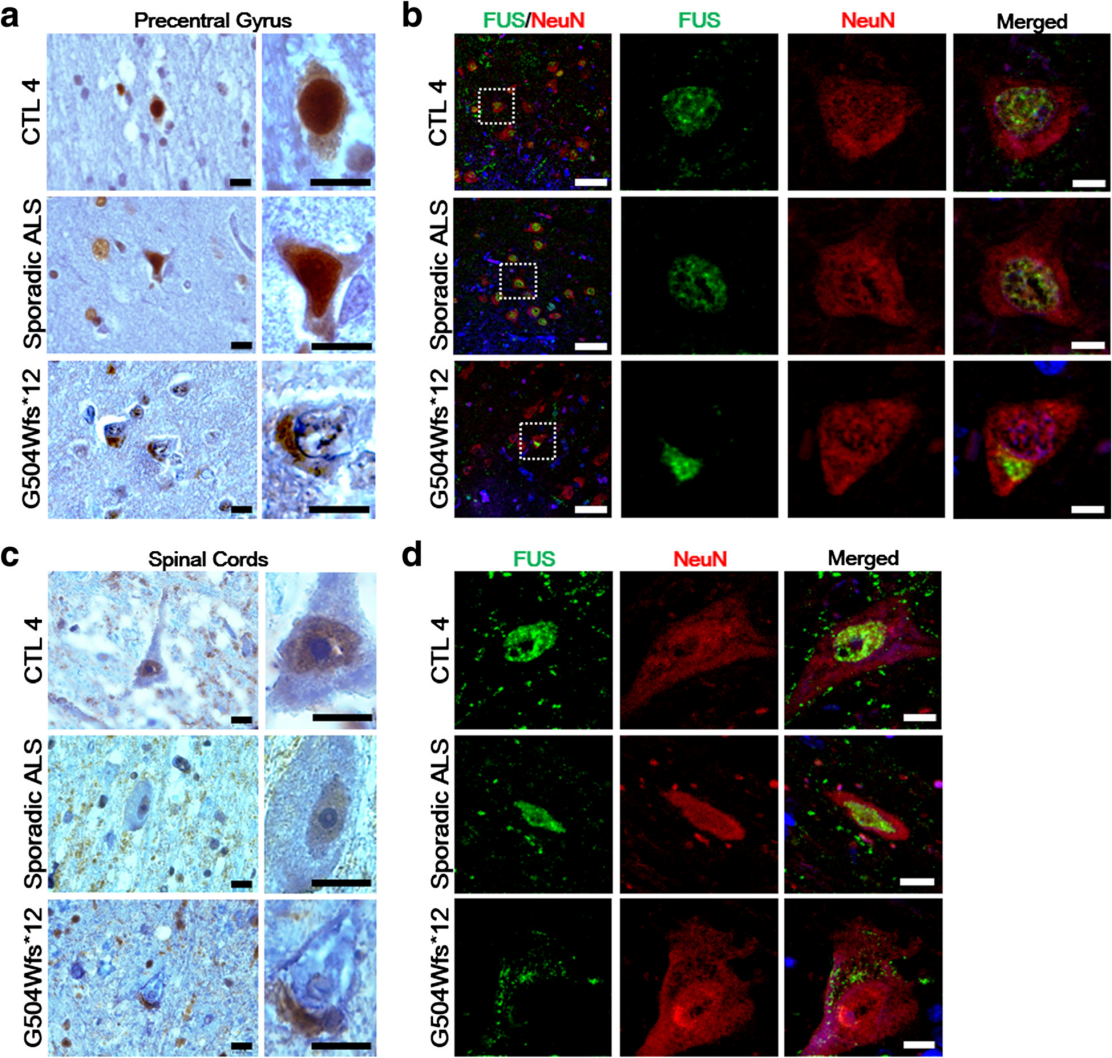
Cytoplasmic incorporation of FUS is present in ALS-FUS patient brain and spinal cords. **a** DAB staining depicts cytoplasmic neuronal inclusions of FUS (as indicated by their morphology) in the precentral gyrus of FUS (p.G504Wfs*12) patient (bottom) compared to the nucleus staining of FUS in a normal control (CTL 4, top) and a sporadic ALS patient (middle). Prominent cytoplasmic or decreased nucleus staining of FUS with ring-like perinuclear inclusions were observed in the motor neurons of the ALS patient. The enlarged images are shown in the right panels. Scale bars = 10 μm. **b** FUS pathology was confirmed by double-label immunofluorescence for FUS (green) and NeuN (red) in a normal control (top), sporadic ALS patient (middle), and FUS (p.G504Wfs*12) patient (bottom). Boxed region in the left panel is enlarged in the right panels. Note that cytoplasmic FUS expressed in a normal control are microglia (Additional file 2: Figure S2). Cells were counter stained with the nuclear marker DAPI (blue). Scale bars = 50 μm for the merged left panels and 7.5 μm for the right panels. **c** The ventral horn of the cervical spinal cord sections from normal control (top), sporadic ALS patient (top), and FUS (p.G504Wfs*12) patient (bottom) were compared. The same pathological features were observed by DAB staining in the spinal cords of the FUS (p.G504Wfs*12) patient. Scale bars = 10 μm. **d** The corresponding sections were processed for double-label immunofluorescence. FUS pathology was confirmed by FUS (green) and NeuN (red) staining. Cells were counter stained with the nuclear marker DAPI (blue). Scale bars = 10 μm

Human autopsy samples were used to reveal the distribution of FUS in the brain and spinal cord of FUS (G504Wfs*12) patient compared to a normal control (CTL 4) and a sporadic ALS patient without any known mutation. Immunohistochemical profiles demonstrated that wild-type FUS was confined predominantly to the nucleus in the majority of neurons in the control brain. A similar distribution of FUS immunoreactivity was also seen in a sporadic ALS patient. In contrast, prominent cytoplasmic or decreased nuclear staining of FUS with ring-like perinuclear inclusions was observed in the FUS (G504Wfs*12) case (Fig. 2a). To confirm cytoplasmic accumulation of FUS in neuronal cells from the FUS (G504Wfs*12) patient, we performed double-label immunohistochemistry for NeuN (neuronal nuclei marker) and FUS. This demonstrated co-labeling of NeuN in the nucleus and the mutant FUS in the cytoplasmic of the same neurons from the ALS-FUS patient, in contrast to the localization of the FUS protein in the nucleus of neurons from healthy control and a sporadic ALS patient (Fig. 2b, Additional file 2: Figure S2).

We also determined the pathogenic features of the mutant FUS in spinal cord motor neurons. Consistent with findings in the precentral motor cortex, immunohistochemistry for FUS in NeuN-positive cells revealed the same pathological feature in the ventral horn of spinal cords (Fig. 2c, d). The sections of spinal cords from both the normal controls and the sporadic ALS patient demonstrated FUS in the nucleus of those neurons. By contrast, mutant FUS were excluded from the nucleus of ALS-FUS patient neurons.

Interestingly, FUS was predominantly nuclear in the postcentral gyrus and dorsal horn neurons of FUS (p.G504Wfs*12) patient indicating that FUS abnormalities are FUS abnormalities are observed in the motor system to a greater extent than that observed for the patient sensory neurons (Additional file 3: Figure S3). This is the first report on the case of FUS (p.G504Wfs*12) pathology on autopsy ALS samples.

### Endogenous mutant FUS pathology in primary patient fibroblasts

**Fig. 3:**
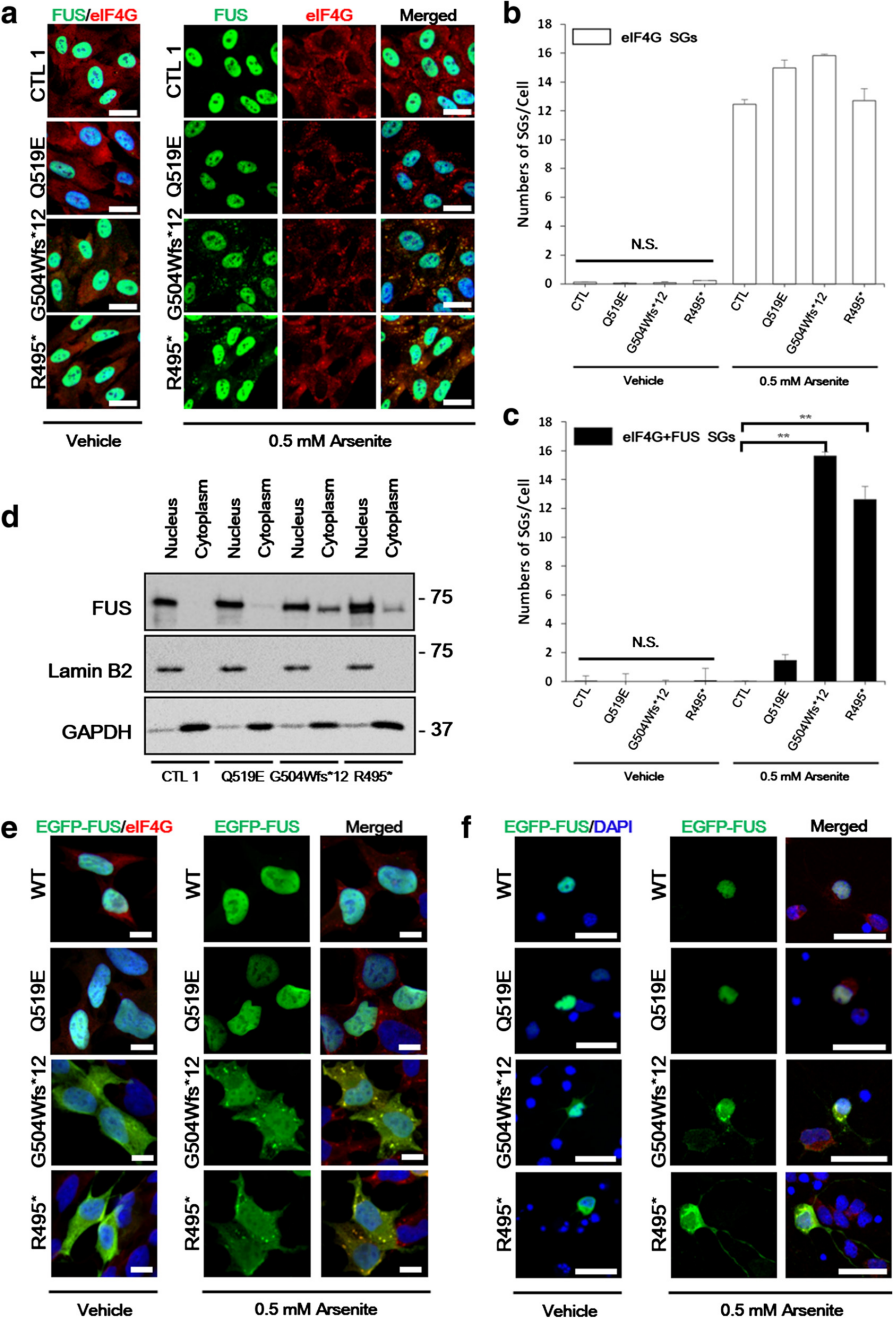
Endogenous FUS is partially mislocalized in patient fibroblasts with G504Wfs*12 and R495* mutations. **a** Primary fibroblasts cultures examined by confocal microscopy. A representative control image shows intense staining for FUS (green) in the nuclei (DAPI) and the stress granule markers eIF4G (red) in the cytoplasm. Patients with the G504Wfs*12 and R495* mutations near the NLS region also show that a majority of FUS protein in the nuclei with a slight increase of cytoplasmic FUS. In response to oxidative stress conditions, cytoplasmic FUS-positive inclusion bodies of G504Wfs*12 and R495* mutation co-localized with eIF4G stress granules (red). Cells were counter stained with the nuclear marker DAPI (blue). Scale bars = 25 μm. Bar graphs represent **b** the numbers of stress granules and **c** the numbers of FUS-positive stress granules (SGs). Data are from three experiments (the mean ± SEM, n = 20). One-way ANOVA followed by Tukey multiple comparisons test; **p < 0.001; N.S., not significant. **d** Cell fractionation analysis of cultured fibroblasts from ALS patients and controls showing an increased cytoplasmic expression of FUS in G504Wfs*12 and R495* patients compared with a representative control and Q519E patient. The upper band of FUS in the nucleus fraction of FUS (p.R495*) patient fibroblasts presumably an allele without a mutation and the lower band indicates the allele with the truncated R495* fragment. Lamin B2 and GAPDH are loading controls for the nuclear and cytoplasmic fractions, respectively. **e** HEK-293 cells were transfected with green fluorescent protein (GFP) wild-type FUS or FUS containing the ALS-associated mutations and treated with vehicle or 0.5 mM arsenite for 30 min. The cells were then processed for immunofluorescence analysis. Localization of GFP-tagged FUS wild type or the indicated FUS mutations (green), eIF4G stress granules (red) are shown. Cytosolic eIF4G co-localizes with FUS aggregates after oxidative stress. GFP (green) and eIF4G (red) show an increased overlap between mutant FUS (p.G504Wfs*12, p.R495*) and eIF4G as compared to wild-type FUS (WT) and eIF4G. Nuclei are shown by DAPI staining. Scale bars = 10 μm. **f** Rat E18 primary cortical neurons were cultured for 21 days and were transfected with constructs expressing wild-type FUS or ALS-associated mutants of FUS (green). After stress, redistribution of mutant FUS aggregates (green) into eIF4G (red) under oxidative stress is demonstrated. Nuclei are shown by DAPI staining. Scale bars = 25 μm.

The residues of the three mutants (p.G504Wfs*12, p.R495*, p.Q519E) are all located in the C-terminal NLS-containing domain of FUS. To examine the presence of ALS-FUS pathology in ALS patient fibroblasts, a punch skin biopsy were obtained from normal controls and ALS patients to isolate their fibroblasts. Primary fibroblasts from healthy individuals (CTL 1, 2, and 3) showed endogenous FUS entirely in the nucleus (Fig. 3a, left panels). Contrary to the report showing endogenous neuronal FUS harboring the G504Wfs*12 or R495* mutation in the cytoplasm with decreased staining in the nucleus [26], we observed more abundant nuclear immunoreactivity of FUS and somewhat diffuse cytoplasmic immunoreactivity on patient-derived fibroblasts that harbor either the G504Wfs*12 or R495* mutation. Surprisingly, FUS (p.Q519E) did not even show any cytosolic mislocalization. These results suggested that either FUS (p.Q519E) does not contribute to the pathogenic potential of ALS or that its mislocalization failed to be captured in the fibroblast model.

Stress agents are known to induce cytoplasmic granules, and various ALS-causing *FUS* mutations have previously been reported to be recruited to those stress granules under stress conditions [21]. Sodium arsenite (referred to as arsenite) is widely used to induce oxidative stress in cells. To determine whether the cytoplasmic FUS protein in the patient fibroblasts could be recruited into stress granules, we stressed cells with arsenite, and observed the shift of dispersed FUS G504Wfs*12 or R495* proteins to cytoplasmic stress granules, which is similar to the response of the eukaryotic translation initiation factor 4G (eIF4G) (Fig. 3a, right panels, and Fig. 3b, c). Again, the Q519E mutant remained in the nucleus under such stress conditions. In addition to oxidative stress induced by sodium arsenite, we tested hyperosmotic stress induced by 0.4 M sorbitol for 1 hr [7]. In response to sorbitol stress, the amount of FUS in the cytoplasm increased with corresponding decrease in the nucleus. Importantly, the accumulation of cytoplasmic FUS granules in mutant fibroblasts is clearly much greater than that in healthy controls (Additional file 4: Figure S4).

Subcellular fractionation of fibroblasts was performed to further investigate the localization of endogenous FUS. In agreement with the immunofluorescence results, the shorter G504Wfs*12 and R495* mutants could be distinguished from the longer wild-type FUS by Western blotting, showing that the mutants were more detectable in the cytosol and that the wild type was exclusively detected in the nucleus. In contrast, the Q519E mutant was detected in only the nucleus (Fig. 3d). These data suggest that patient-derived fibroblasts may not fully reflect the ALS pathology with disease-associated mutations in FUS.

### Mutant FUS pathology in transfected HEK-293 cells and primary neurons

We aimed to examine whether the similar mutant FUS characteristics of patient fibroblasts, carrying the Q519E mutation, i.e., predominant nucleus FUS staining, was also observed in transfected cells. We overexpressed the cDNA encoding an N-terminal green fluorescence protein (GFP)-tagged wild-type or a mutant FUS in HEK-293 cells. The transiently transfected G504Wfs*12 and R495* mutants showed both nuclear and cytosolic distribution, whereas the Q519E mutant like the wild-type FUS resided predominantly in the nucleus (Fig. 3e, left panels). To determine whether the cytoplasmic mutant FUS could be incorporated into stress granules under oxidative stress conditions, we exposed the cells to arsenite. Both the G504Wfs*12 and R495* mutants showed the incorporation of their cytoplasmic FUS into eIF4G-containing granules, but the Q519E mutant still behaved like the wild-type FUS (Fig. 3e, right panels).

The neuropathology of ALS is characterized by degenerating neurons in the brain and spinal cord, which is coincident with neuronal cytoplasmic inclusions of ALS-associated FUS proteins [27]. To determine the distribution of wild-type or mutant FUS constructs in neurons, we cultured cortical neurons from rats on embryonic day 18 rats and transfected them with GFP-tagged FUS constructs. The neurons were first cultured for 21 days and then transfected for 48 hrs before fixation. As shown in HEK-293 cells, both G504Wfs*12 and R495* mutants resided largely in the cytosol, which is contrary to the patterns that were observed in patient fibroblasts (Fig. 3f, left panels). When rat cortical neurons were exposed to oxidative stress, the cytosolic FUS (p.G504Wfs*12 and p.R495*) was further incorporated in eIF4G-positive stress granules (Fig. 3f, right panels). Interestingly, both the Q519E mutant and the wild-type FUS continued to reside in the nucleus before and after stress induction. These findings suggest that neurons from murine models may fail to reflect certain neuronal pathologies in human ALS-FUS brain or spinal cord samples. Moreover, overexpressed FUS may also cause deleterious effects that may be unrelated to ALS pathologies in transfected cells [28].

### Endogenous mutant FUS that recapitulates autopsied ALS pathology is iNeuron-specific

**Fig. 4:**
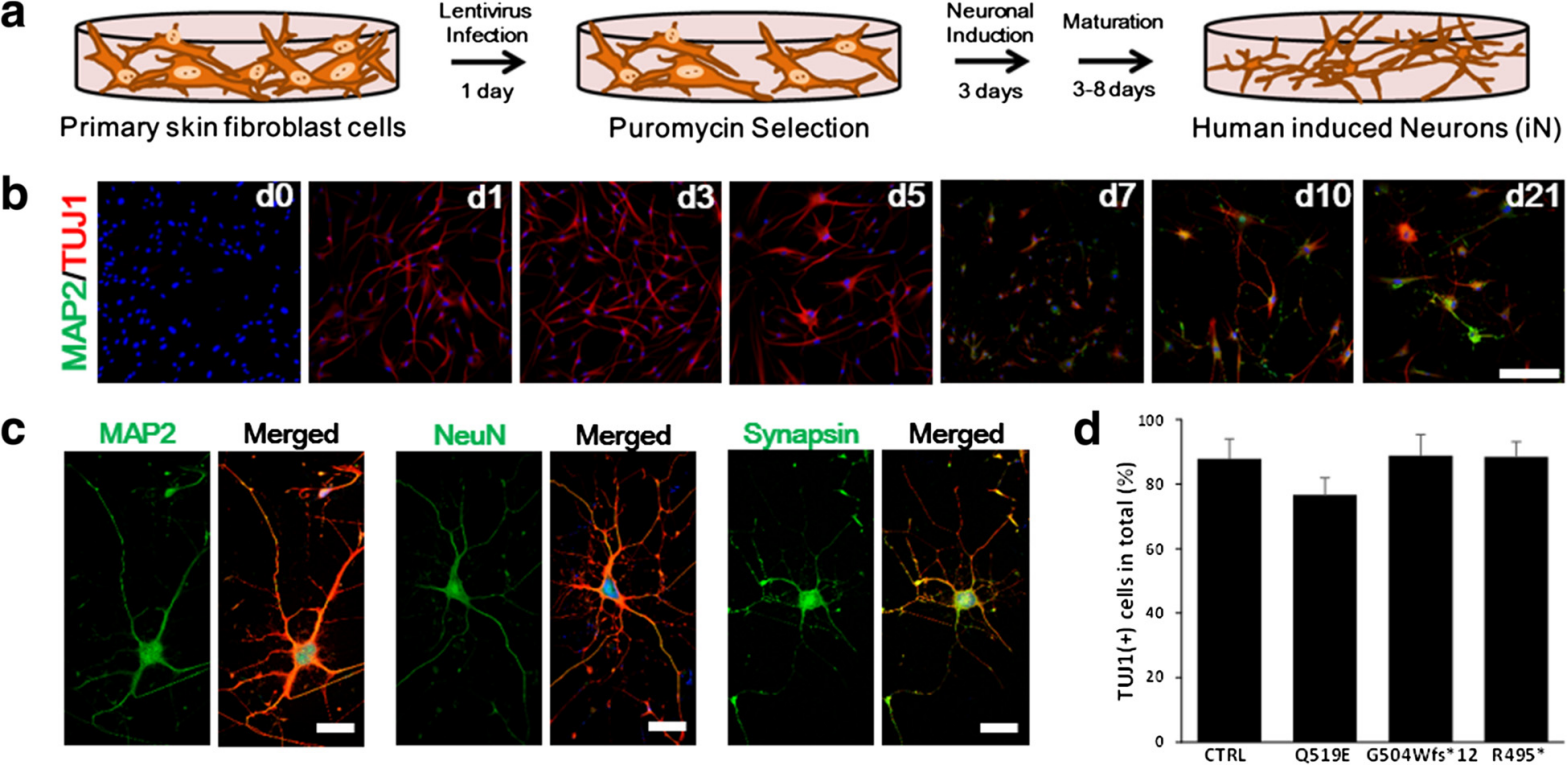
Direct conversion. **a** Schematic of the experimental protocol. **b** Cells probed with a mature neuronal marker anti-MAP2 (green) and a tubulin marker anti-TUJ1 (red) revealed that mature iNeurons are detected from day 7. Cells were counter stained with the nuclear marker DAPI (blue). Scale bars = 250 μm. **c** Expression of mature neuronal markers in iNeurons. Green: MAP2, NeuN, Synapsin; red: TUJ1; blue: DAPI. Scale bars: 50 μm. **d** Quantification of iNeurons based on TUJ1-positive cells divided by the number of initial plating cells in response to PTBP1 shRNA. Cells untreated with shPTBP1 had no TUJ1-positive staining. Data are from three experiments (the mean ± SEM, *n* = 30–82).

To develop more accurate disease models for ALS, we trans-differentiated ALS patient fibroblasts into induced neurons (iNeurons) by repressing a single RNA binding polypyrimidine-tract-binding (PTB) protein. To generate human iNeurons, we infected both patient and control fibroblasts with a lentivirus-repressing PTBP1, according to and modified from our recently published methods [16]. The subsequent culture conditions are provided in the schematic overview in Fig. 4a. In confocal cellular immunostaining assays, cells exhibited typical neuronal morphology, and nearly all cells were strongly positive for TUJ1 (the early neuronal marker βIII-tubulin). Within a day of neuronal induction, the cells were positive for TUJ1, and from 5–21 days, an increase in MAP2 (neuronal dendrites marker) immunostaining was observed (Fig. 4b). The maturated morphology of iNeurons with dendritic branching were confirmed with MAP2, NeuN (neuronal nuclei marker), and synapsin (neuronal synapsis marker) immunostaining at day 10 of neuronal induction (Fig. 4c). The percentage of neuronal tubulin marker TUJ1-positive iNeuron cells of the controls and three ALS patients with different types of *FUS* mutations were similar (Fig. 4d).

**Fig. 5:**
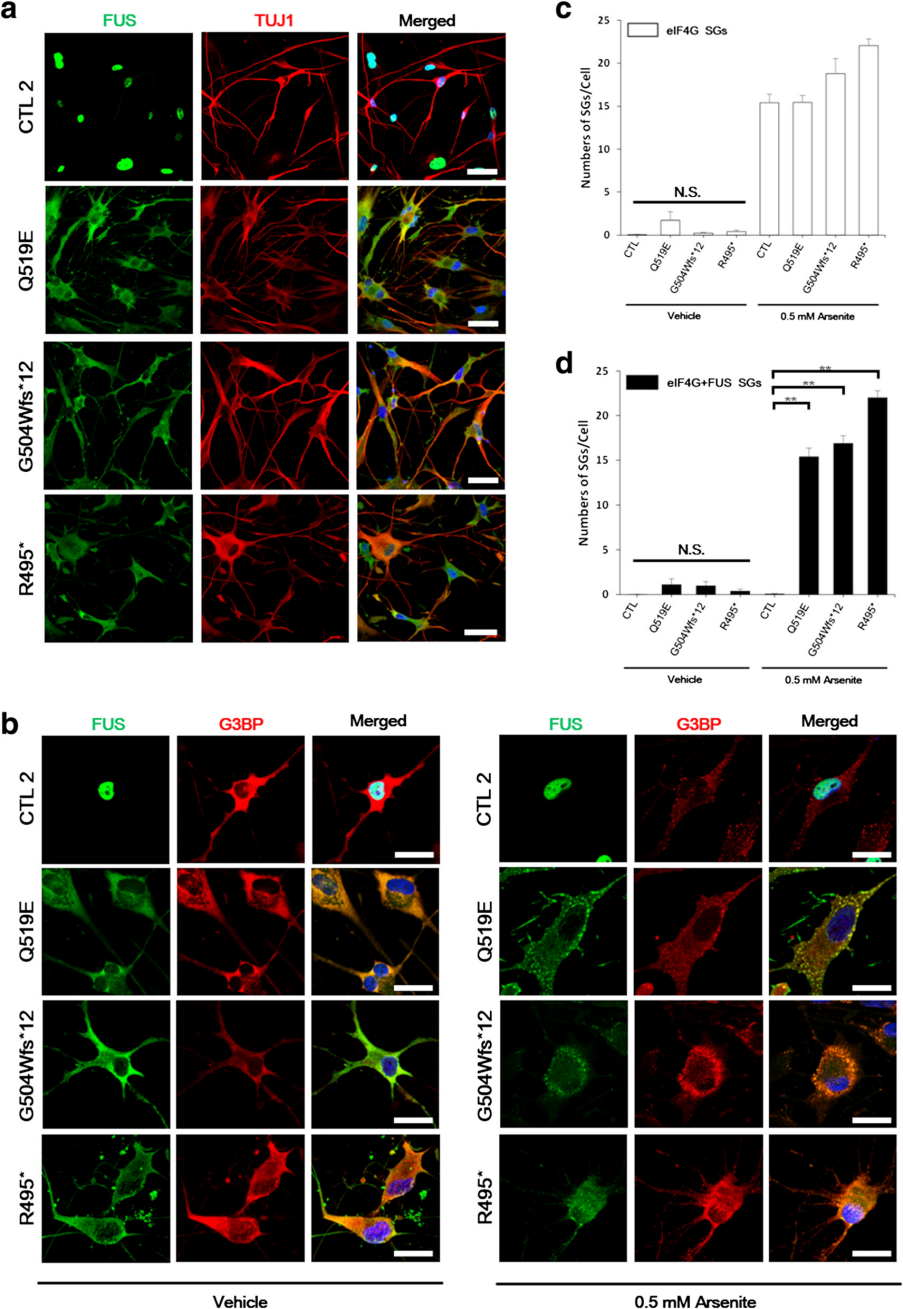
Endogenous FUS is mislocalized to the cytoplasm and is incorporated into cytoplasmic stress granules in response to arsenite in patient iNeurons. **a** A representative control shows intense staining for FUS (green) in the nuclei (DAPI) in TUJ1-positive (red) iNeurons at day 10 of neuronal induction, whereas the patients show a majority of FUS protein in the cytoplasm. Cells were counter stained with the nuclear marker DAPI (blue). Scale bars = 50 μm. **b** Confocal images of vehicle treated iNeurons (left panel) as compared to cells treated with 0.5 mM arsenite for 30 min (right panel) at day 10 are shown. A representative control shows FUS protein predominantly localized to the nuclei. ALS-FUS patient with Q519E mutation recapitulated the FUS neuropathology only in iNeurons: iNeurons from the patient show a majority of FUS protein (green) in the cytoplasm. In response to oxidative stress conditions, cytoplasmic FUS-positive inclusion bodies (green) in iNeurons were co-localized with G3BP stress granules (red). Cells were fixed and probed by immunofluorescence for DAPI (blue). Scale bars = 25 μm. Bar graphs represent (**c**) the numbers of stress granules and (**d**) the numbers of FUS-positive stress granules (SGs). Data are from three experiments (the mean ± SEM, *n* = 20). One-way ANOVA followed by Tukey multiple comparisons test; **p < 0.001; N.S., not significant

In control iNeurons, endogenous FUS was predominantly nuclear (Fig. 5a and b left panels). In contrast, the patient iNeurons of G504Wfs*12 or R495* exhibited reduced endogenous FUS immunoreactivities in the nucleus along with increased cytoplasmic FUS. Considering that FUS was predominantly distributed in the nucleus of patient fibroblasts, FUS expression in iNeuron models seem to more closely mirror the FUS neuropathology found in ALS patients than those observed in patient fibroblasts. Intriguingly, the FUS (p.Q519E) patient also showed cytoplasmic localization of FUS with less nuclear distribution in the iNeuron model.

To determine whether the cytosolic FUS (p.Q519E) could be induced to stress granules in iNeurons, we treated iNeurons with arsenite, and in line with the results with the cytoplasmic FUS in G504Wfs*12 or R495* patient iNeurons, we observed co-localization of the FUS (p.Q519E) mutant with arsenite-induced stress granules, which was further validated by the detection of the colocalization of the Ras-GTPase-activating protein SH3 domain binding protein (G3BP), another known component of stress granules (Fig. 5b, right panels). Co-localization of the cytosolic FUS inclusions with eIF4G under oxidative stress was also confirmed and quantified (Additional file 5: Figure S5, and Fig. 5c, d). These findings suggest that unlike patient-derived fibroblasts and transfected cell models, only patient iNeurons are able to fully capture the neuropathology of *FUS* mutations with a disrupted NLS region.

## Conclusions

Mutations in *FUS* have been strongly implicated as the genetic cause of ALS [2, 29]. In this study, we performed functional analysis of three different *FUS* mutations found in ALS patients, including the two *de novo* mutations (p.G504Wfs*12, p.R495*) we previously identified by trio study in sporadic ALS [30] and a novel variant (p.Q519E) by multi-gene panel testing in familial ALS (Table 1). All these mutations were located in the C-terminal region that contains the nuclear localization signal (NLS). FUS accumulation in neuronal cytoplasmic inclusions along with a degree of nuclear clearance are histopathological hallmarks of patients with FUS-mediated ALS, especially for the mutations located at the NLS region [2, 31]. Consistently, we show for the first time that FUS (p.G504Wfs*12) exhibited the accumulation of cytoplasmic FUS and the depletion of nuclear FUS in patient brain and spinal cord motor neurons. The autopsy results demonstrated typical ALS-FUS features of cytoplasmic aggregation and nuclear clearance of FUS in neurons, which have also been described in the autopsy of patients with other FUS mutations in the NLS region.

As of now, cultured patient fibroblasts have been used as cellular models for disease studies. Induced pluripotent stem cell (iPSC)-derived neurons from patients with a *FUS* mutation appear to provide a suitable model for understanding pathophysiological mechanisms of *FUS* mutations; however, one of the problems in skin fibroblast models is that some common FUS-associated pathological hallmarks found in autopsy cases are not consistently identified in patient fibroblasts [13]. Although iPSC-based models are useful in identifying the molecular and cellular defects in neuronal abnormality and instrumental for in vitro drug screening for therapeutic effects, the process of generating iPSC-derived neurons from human fibroblasts is intricate. To develop disease models more efficiently, we directly converted the fibroblasts from patients with *FUS* mutations into induced neuron (iNeuron) by repressing a polypyrimidine-tract-binding (PTB) protein. As PTB is naturally down-regulated during neuronal induction in development, PTB regulation enhanced the neurogenesis program in the fibroblasts [16]. As shown in the present data, iNeuron is a rapid and highly disease-relevant cell model. Compared to the majority of nuclear FUS distribution in patient fibroblasts carrying mutations in the NLS region, iNeurons demonstrated a clear increase in cytoplasmic distribution and a concurrent decrease in the nuclear distribution of mutant FUS. Moreover, cytosolic aggregates of FUS could be induced under oxidative stress conditions. The analysis on iNeurons from a FUS (p.G504Wfs*12) patient recapitulated all key features of FUS pathology found in the patient brain and spinal cord motor neurons, thus confirming that iNeurons as a more disease-relevant in vitro model that accurately mirrors disease pathology of the patient. Intriguingly, the FUS (p.Q519E) patient who had endogenous FUS distributed in only the nucleus in fibroblast models or transiently transfected cells demonstrated a cytosolic mislocalization and aggregation of FUS only in the iNeuron model. These findings further support this new model as a useful research tool for studying ALS-FUS pathogenesis.

FUS proteinopathies in ALS neuronal degeneration have been poorly understood due to the lack of clinically relevant cell models for the disease. The identification of disease-causing genes and the development of patient-specific and disease-relevant cell models for functional analysis are critical for advancing our understanding of the pathophysiology in ALS. Studies using patient iNeurons may reveal additional features of FUS pathology in the cytoplasm that may have escaped previous studies on patient fibroblasts [11]. Similarly, mutant FUS cDNA constructs of patients whose fibroblasts or each cDNA construct does not display typical FUS pathology may have distinct pathologic features, which can now be dissected in iNeurons.

ALS-FUS patient fibroblast models present endogenous cytoplasmic FUS incorporation into stress granules; however, FUS in patient fibroblasts are predominantly expressed in the nucleus. Murine neurons transiently transfected with mutant FUS constructs revealed both decreases in the nucleus and increases in the cytosol, and upon stress, cytosolic FUS could be induced into stress granules. Yet, murine neurons may be insufficient to capture all key mechanism in neuronal pathology in human brain or spinal cords.

Development of more disease-relevant experimental models from ALS patients that recapitulate the characteristics of neuronal dysfunction found in human post-mortem tissues will open new doors to both understanding pathophysiologic mechanisms in ALS-FUS and developing new therapeutic strategies. Therefore, simple, reliable, and reproducible iNeuron models are promising in that they may greatly accelerate ALS research.

## Methods

### Subjects

Three ALS patients with different types of *FUS* mutations were enrolled in this study. We have recently identified *FUS* mutations (p.G504Wfs*12, p.R495*, and p.Q519E) by direct sequencing and multi-gene panel testing [17, 19, 32]. These patients showed onset at age 27 to 34 with various disease progression. Skin fibroblasts were obtained from these ALS patients with disrupted NLS region and three healthy controls. Autopsy tissues were obtained from two patients: one ALS-FUS patient (p.G504Wfs*12) and one sporadic ALS patient without any known mutation in *FUS*, *C9orf72*, *SOD1*, *ALS2*, *SPG11*, *UBQLN2*, *DAO*, *GRN*, *SQSTM1*, *SETX*, *MAPT*, *TARDBP*, and *TAF15*. The clinical and genetic findings are summarized in Table 1. The study protocol was approved by the Institutional Review Board of Hanyang University Hospital, and written informed consents were obtained from all patients involved in the study (IRB# 2011-R-63).

### Structural modelling

For a structural analysis, we sought for an applicable protein structure in PDB (ID: 4FDD), which contains Transportin-1 and FUS domains. Because the FUS domain includes the Q519 residue, the influence of Q519E mutation on the complex can be examined. The PDB complex consists of Transportin-1 (chain A: residue number from 371 to 890) and FUS (chain B: residue number from 498 to 526). The missing part (residue number from 321 to 370) and N-terminal region (from 1 to 320) in Transportin-1 was removed from the original PDB structure because they are not relevant to direct interactions with the FUS domain. The FUS missing residues from 498 to 506 were generated and minimized to find their local minima with keeping the rest atomic coordinates unchanged. To examine the effect of Q519E mutation on the FUS-Transportin-1 binding, a hydrogen bonding analysis was performed between FUS and Transportin-1 structures. Because the structure has no hydrogen atoms, we used an implicit hydrogen bonding analysis with the following loose criteria, the bond distance below 5 Å between acceptor and donor atom and the angle above 90°, among acceptor, donor, and the prior atom connected to the donor atom. The analysis was performed in CHARMM (Chemistry at Harvard Macromolecular Mechanics) [33], and the structure was visualized using Jmol (an open-source Java viewer for chemical structures in 3D. http://www.jmol.org/)

### Immunohistochemistry and immunofluorescence

Autopsied samples of brain and spinal cord were obtained from one ALS-FUS patient (p.G504Wfs*12), one sporadic ALS patient, and one healthy control. Immunohistochemistry was performed on 5 μm thick paraffin sections. Tissues were deparaffinized, rehydrated in serial changes of xylene and ethanol gradients and autoclaved for 10 min in 10 mM citric acid, pH 8.0. Sections were then blocked with 10 % normal goat serum (vol/vol) in PBS. For immunostaining, rabbit polyclonal antibodies reactive to FUS (Abnova) were applied on the precentral motor cortex and postcentral gyrus, and mouse antibodies against FUS (Proteintech) were used on spinal cord tissue. The sections were colorimetrically developed using the 3,3’-diaminobenzidine DAB substrate kit (Vector Labs) for 1 min and counter stained with haematoxylin (Sigma-Aldrich), dehydrated, and coverslipped in Permount medium. Images were acquired with a Leica DM5000B microscope.

For double labeling immunofluorescence, paraffin-embedded sections were blocked with 10 % normal goat serum (vol/vol). The primary antibodies used were mouse antibodies against FUS (Proteintech) and rabbit polyclonal antibodies against NeuN (Millipore), GFAP (Dako), and Iba-1 (Wako). The secondary antibodies included Alexa Fluor 488-conjugated mouse and tetramethylrhodamine B isothiocyanate (TRITC)-conjugated rabbit antibodies. Images were acquired using a Leica TCS SP5 confocal microscope.

### Plasmids and site-directed mutagenesis

N-terminally GFP-tagged wild-type human FUS cDNA was cloned into the pReceiver vector (Genecopoeia). To make the mutant DNA (p.Q519E, p.G504Wfs*12, p.R495*), *in vitro* mutagenesis of the GFP-tagged FUS cDNA was conducted using the EZchange™ site-directed mutagenesis kit (Enzynomics) according to the manufacturer’s protocol.

### Cell culture and reagents

HEK-293 cells were cultured in Dulbecco’s modified Eagle’s medium (DMEM) supplemented with 10 % fetal bovine serum (Gibco), sodium bicarbonate, sodium pyruvate (Sigma-Aldrich), and antibiotics. Primary rat neurons were maintained in Neurobasal medium supplemented with 2 % (vol/vol) B27, 1 % (vol/vol) GlutaMAX, 100X insulin-transferrin-selenium (ITS) (all from Invitrogen), and antibiotics.

HEK-293 and primary rat neurons were transiently transfected with GFP-tagged wild-type or mutant human *FUS* cDNA using Lipofectamine 2000 (Invitrogen) according to the manufacturer’s instructions. After 48 hrs, the cells were fixed in the presence or absence of stress for immunofluorescence staining as described below.

For oxidative stress induction, vehicle (water) or 1 M stock solution of sodium arsenite (Sigma-Aldrich) dissolved in water was added to the media at a final concentration of 0.5 mM for up to 30 min. For hyperosmotic stress induction, vehicle (growth media) or 0.4 M sorbitol (Sigma-Aldrich) dissolved directly into the growth media for up to 1 hr.

### Conversion of human skin fibroblasts to iNeurons

Fibroblasts were obtained from forearm skin with a punch biopsy (Table 1). Fibroblasts were cultured and maintained in DMEM supplemented with 20 % FBS, non-essential amino acids (all from Gibco), sodium bicarbonate (Sigma-Aldrich), and 1 % (vol/vol) Penicillin/Streptomycin/Fungizone (Cellgro). In all experiments, passage-matched fibroblasts (passages 3–5) were used. Fibroblast were seeded at a density of 1 × 10^4^ cells/cm^2^ and used for experiments after cell synchronization by serum starvation at matched time points.

For direct conversion, human fibroblasts were seeded onto matrigel (BD Biosciences)-coated 24-well tissue culture dishes or cell culture flasks (Nunc). Induced neurons (iNeurons) were generated from patient-derived fibroblasts using lentiviral transduction of the shRNAs against human PTBP1 (Sigma-Aldrich MISSION) according to our previously described protocol [16, 34]. Thirty hours after the shRNA treatment, the cells were selected with 1 μg/ml puromycin for another 30 hrs. Selected cells were replaced for 3 days in N3 media (DMEM/F12 (Gibco) supplemented with 25 μg/ml Insulin, 50 μg/ml apo-transferrin, 20 nM progesterone, 100 nM putrescine, and 30 nM sodium selenite (all Sigma-Aldrich)), 10 ng/ml bFGF (Gibco), supplemented with BDNF, CNTF, GDNF, and NT3 (all PeproTech) as previously described [16]. From day 4 to the day of analysis, the cells were maintained in N3 media supplemented with 2 % FBS.

### Immunocytochemistry and confocal microscopy

Fibroblasts, HEK-293, primary rat neurons, and iNeurons were washed with 1 × PBS, fixed with 4 % paraformaldehyde (PFA) for 15 min at room temperature and then washed three more times with PBS. Cells were permeabilized by incubation in 0.3 % Triton X-100 for 10 min at room temperature, washed with PBS, and then blocked for 1 hr in 5 % normal goat serum (Vector Labs). Cells were incubated with primary antibodies for 2 hrs at room temperature, washed three times with 1 × PBS, and incubated with secondary antibodies for 1 hr at room temperature. After three additional washings with 1 × PBS, nuclei were stained with DAPI. Coverslips were mounted on glass slides with Fluoromount-G (SouthernBiotech). The primary antibodies used included mouse monoclonal antibodies against C-terminus FUS (Santa Cruz Biotechnology), FUS (Proteintech), G3BP (BD Transduction Laboratories), and rabbit polyclonal antibodies against eIF4G (Santa Cruz Biotechnology), FUS (Abnova). For neuronal cell markers, mouse monoclonal antibody reactive to β-tubulin III (TUJ1; Covance) and rabbit polyclonal antibody to MAP2 (Cell Signaling Technology), NeuN (Millipore), and Synapsin I (Chemicon) were used. Secondary antibodies were Alexa Fluor 488-conjugated and/or TRITC-conjugated mouse or rabbit antibodies (Gibco). Images were acquired with a Leica TCS SP5 confocal microscope. The stress granules were counted manually. Twenty cells from each patient fibroblasts or iNeurons were chosen based on DAPI staining of nuclei (*n* = 3). Significance between stress granule formations was calculated using one-way ANOVA followed by Tukey multiple comparisons test.

### Nuclear-cytoplasmic fractionation and immunoblot analysis

Cell fractionation was performed using the NE-PER Nuclear and Cytoplasmic Extraction Reagents kit (Thermo Fisher Scientific) according to the manufacturer’s protocol. Nuclear and cytoplasmic extracts from fibroblasts were analyzed by Western blotting. Equal amounts of protein from each sample were separated by 10 % sodium dodecyl sulfate polyacrylamide gel electrophoresis (SDS-PAGE) and transferred to a PVDF membrane (GE Healthcare). Membranes were blocked with 5 % skim milk. The primary antibodies used were mouse monoclonal antibodies against Lamin B2 (AbCam) and rabbit polyclonal antibodies against FUS (Abnova) and GAPDH (Santa Cruz Biotechnology). The membranes were probed with horseradish peroxidase-conjugated secondary antibodies (Santa Cruz Biotechnology) and developed using West-Q Chemiluminescent Substrate Plus Kits (GenDEPOT).

## Additional files

Additional file 1: Figure S1. Structures of FUS-Transportin-1 complexes. (a) Overall structure of FUS-Transportin-1 complexes are presented in blue sphere and white surface, respectively. The position of FUS (p.Q519) is marked by red sphere models. (b) The focused view around the mutation (p.Q519). The structure of FUS and Transportin-1 complexes are consisted of a ball-and-stick representation. Stick models are colored by atom (N: blue, O: red, C: gray, respectively). The important position of the mutation (p.Q519) is depicted in a red ball-and-stick representation. A possible hydrogen bonding between Q519 of FUS and E509 of Transportin-1 is shown in the red circle with the acceptor-donor distance (3.21 Å). (PDF 69 kb) Additional file 2: Figure S2. FUS is distributed in the cytoplasm in microglia but is absent in astrocytes. FUS (green) is (a) apparently not expressed in GFAP-positive astrocytes, (red) and is (b) cytoplasmic in Iba-1-positive microglia (red, arrows) in the precentral gyrus of a normal control (CTL 4, top), sporadic ALS patient (middle), and FUS (p.G504Wfs*12) patient (bottom). Boxed region in the left panel is enlarged in the right panels. Cells were counter stained with the nuclear marker DAPI (blue). Scale bars = (a) 50 μm for the merged left panels and 10 μm for the right panels, and (b) 25 μm. Cells were counter stained with the nuclear marker DAPI (blue). (PDF 594 kb) Additional file 3: Figure S3. FUS is distributed in the nucleus in ALS-FUS patient postcentral gyrus and dorsal horn. (a) DAB staining depicts predominant nucleus localization of FUS (as indicated by their morphology) in the postcentral gyrus of a normal control (CTL 4, top), sporadic ALS patient (middle), and FUS (p.G504Wfs*12) patient (bottom). The enlarged images are shown in the right panels. Scale bars = 10 μm. (b) The dorsal horn of the spinal cord sections from normal control (top), sporadic ALS patient (top), and FUS (p.G504Wfs*12) patient (bottom) were compared. The same predominant nucleus staining of FUS were observed by DAB staining in the dorsal horn neurons (as indicated by their morphology) of a normal control (top), sporadic ALS patient (middle), and FUS (p.G504Wfs*12) patient (bottom). Scale bars = 10 μm. (PDF 378 kb) Additional file 4: Figure S4. Endogenous FUS is partially redistributed to the cytoplasm in response to sorbitol. Primary fibroblasts of a representative control and the patient with the Q519E mutation shows intense staining for FUS (green) in the nuclei (DAPI) and the stress granule markers eIF4G (red) in the cytoplasm. Patients with the G504Wfs*12 and R495* mutations also show that a majority of FUS protein in the nuclei with a slight increase of cytoplasmic FUS (left panel). Cells treated with 0.4 M sorbitol for 1 hr are shown on the right panel. In response to sorbitol stress, slight decrease of nucleus FUS and increase of cytoplasmic FUS-positive inclusion bodies co-localized with eIF4G stress granules were observed. The accumulation of cytoplasmic FUS granules in mutant fibroblasts were much greater than that in healthy controls. Cells were counter stained with the nuclear marker DAPI (blue). Scale bars = 10 μm. (PDF 821 kb) Additional file 5: Figure S5. Endogenous FUS cytoplasmic incorporation into stress granule marker eIF4G in response to arsenite in patient iNeurons. Immunocytochemistry performed on vehicle treated iNeurons (left panel) as compared to cells treated with 0.5 mM arsenite for 30 min (right panel) at day 10 are shown. A representative control shows FUS protein predominantly localized to the nuclei. All three ALS-FUS patients show a majority of FUS protein (green) in the cytoplasm of iNeurons. Cytoplasmic FUS-positive inclusion bodies (green) were detectable in eIF4G-positive stress granules (red) in patients. Cells were fixed and probed by immunofluorescence for DAPI (blue). Scale bars = 25 μm. (PDF 751 kb)

## Abbreviations

ALS: Amyotrophic lateral sclerosis
ALS-FUS: ALS-associated FUS
eIF4G: Eukaryotic translation initiation factor 4G
FUS: Fused in sarcoma
G3BP: Ras-GTPase-activating protein SH3 domain binding protein
iNeurons: Induced neurons
iPSC: Induced pluripotent stem cell
NLS: Nuclear localization signal
PTB: Polypyrimidine-tract-binding
