# Supplementary figures and images for: Directly converted patient-specific induced neurons mirror the neuropathology of FUS with disrupted nuclear localization in amyotrophic lateral sclerosis
Source: Mol Neurodegener. 2016 Jan 22;11:8. doi: 10.1186/s13024-016-0075-6 (PMC4722778; doi:10.1186/s13024-016-0075-6)

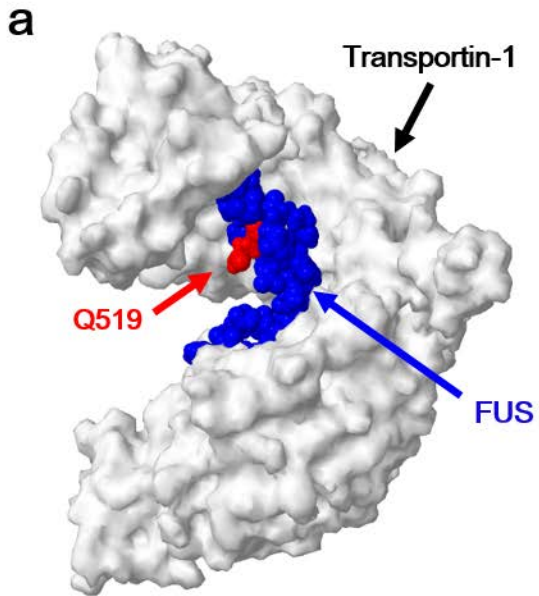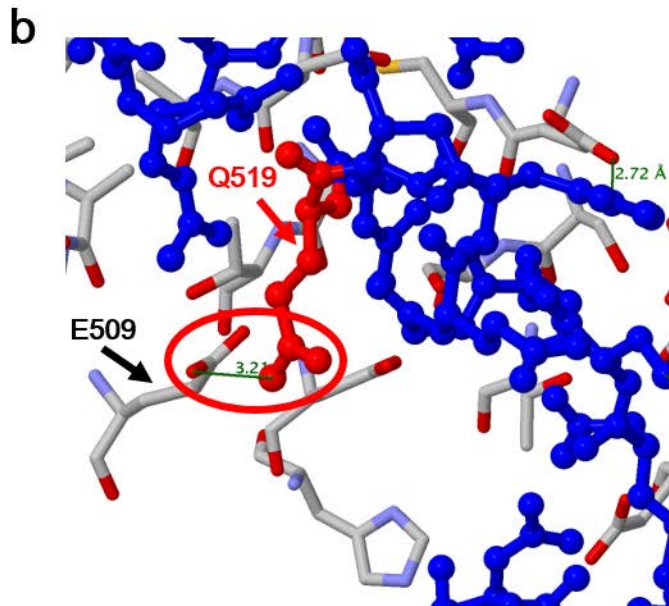

Supplement: Additional file 1: Figure S1. — Structures of FUS-Transportin-1 complexes. (a) Overall structure of FUS-Transportin-1 complexes are presented in blue sphere and white surface, respectively. The position of FUS (p.Q519) is marked by red sphere models. (b) The focused view around the mutation (p.Q519). The structure of FUS and Transportin-1 complexes are consisted of a ball-and-stick representation. Stick models are colored by atom (N: blue, O: red, C: gray, respectively). The important position of the mutation (p.Q519) is depicted in a red ball-and-stick representation. A possible hydrogen bonding between Q519 of FUS and E509 of Transportin-1 is shown in the red circle with the acceptor-donor distance (3.21 Å). (PDF 69 kb) [file 13024_2016_75_MOESM1_ESM.pdf]

**a**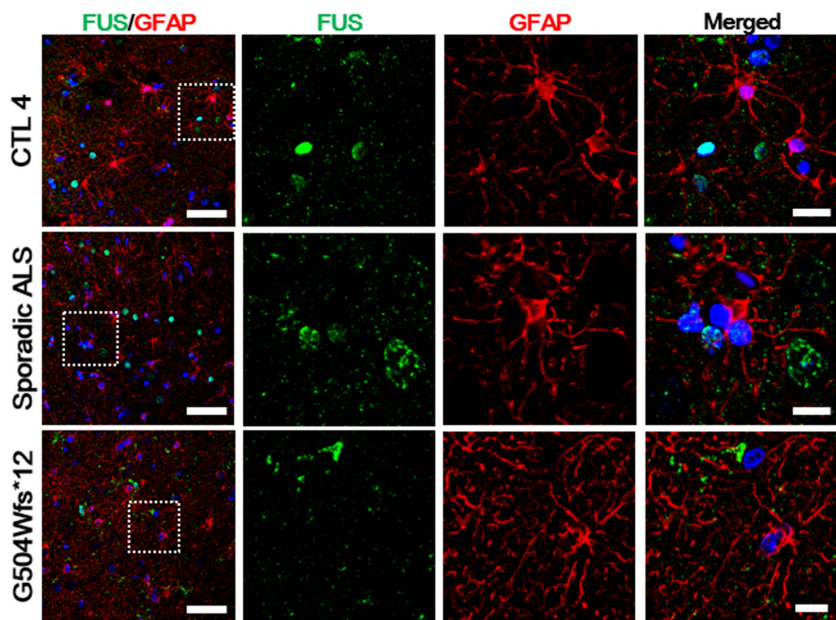**b**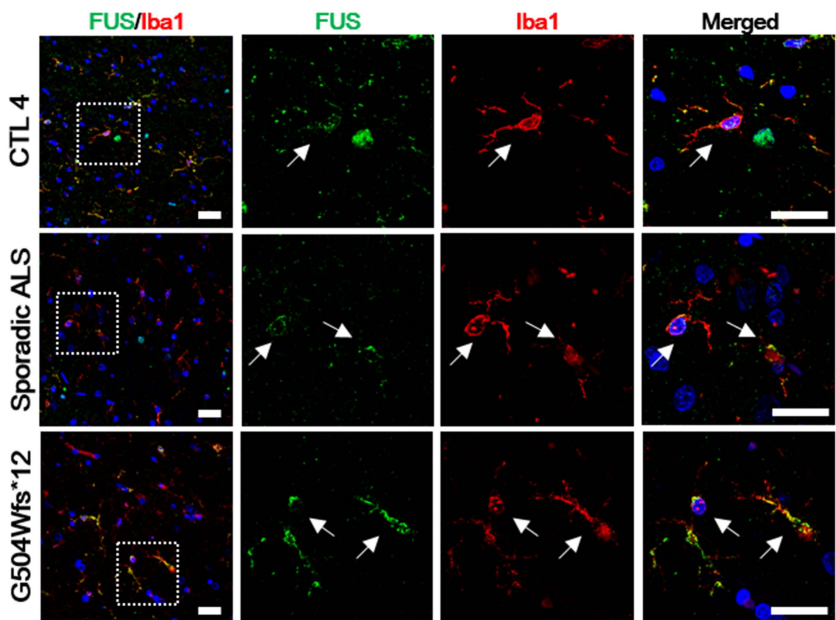

Supplement: Additional file 2: Figure S2. — FUS is distributed in the cytoplasm in microglia but is absent in astrocytes. FUS (green) is (a) apparently not expressed in GFAP-positive astrocytes, (red) and is (b) cytoplasmic in Iba-1-positive microglia (red, arrows) in the precentral gyrus of a normal control (CTL 4, top), sporadic ALS patient (middle), and FUS (p.G504Wfs*12) patient (bottom). Boxed region in the left panel is enlarged in the right panels. Cells were counter stained with the nuclear marker DAPI (blue). Scale bars = (a) 50 μm for the merged left panels and 10 μm for the right panels, and (b) 25 μm. Cells were counter stained with the nuclear marker DAPI (blue). (PDF 594 kb) [file 13024_2016_75_MOESM2_ESM.pdf]

**a****Postcentral Gyrus**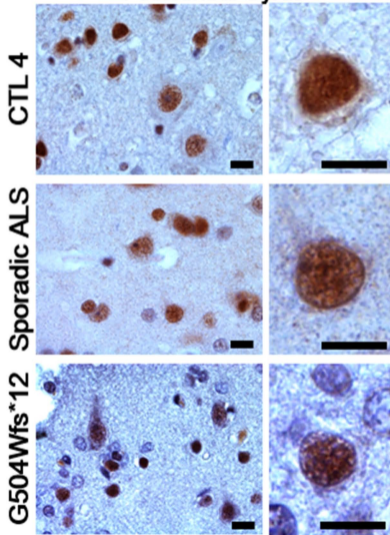**b****Dorsal Horn**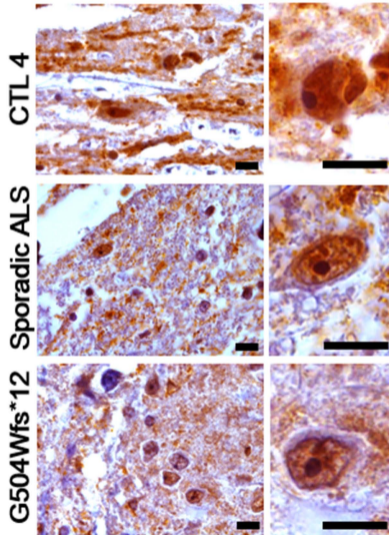

Supplement: Additional file 3: Figure S3. — FUS is distributed in the nucleus in ALS-FUS patient postcentral gyrus and dorsal horn. (a) DAB staining depicts predominant nucleus localization of FUS (as indicated by their morphology) in the postcentral gyrus of a normal control (CTL 4, top), sporadic ALS patient (middle), and FUS (p.G504Wfs*12) patient (bottom). The enlarged images are shown in the right panels. Scale bars = 10 μm. (b) The dorsal horn of the spinal cord sections from normal control (top), sporadic ALS patient (top), and FUS (p.G504Wfs*12) patient (bottom) were compared. The same predominant nucleus staining of FUS were observed by DAB staining in the dorsal horn neurons (as indicated by their morphology) of a normal control (top), sporadic ALS patient (middle), and FUS (p.G504Wfs*12) patient (bottom). Scale bars = 10 μm. (PDF 378 kb) [file 13024_2016_75_MOESM3_ESM.pdf]

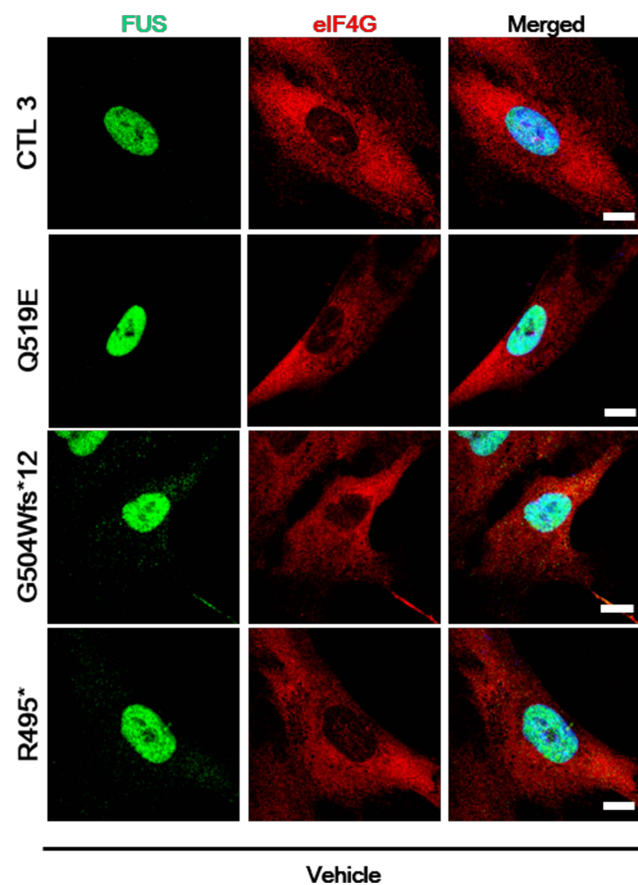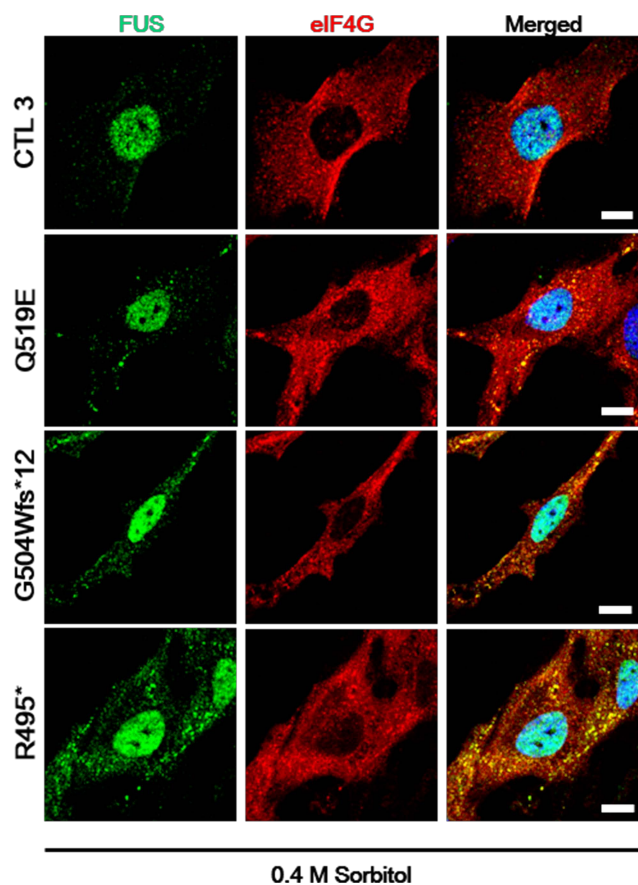

Supplement: Additional file 4: Figure S4. — Endogenous FUS is partially redistributed to the cytoplasm in response to sorbitol. Primary fibroblasts of a representative control and the patient with the Q519E mutation shows intense staining for FUS (green) in the nuclei (DAPI) and the stress granule markers eIF4G (red) in the cytoplasm. Patients with the G504Wfs*12 and R495* mutations also show that a majority of FUS protein in the nuclei with a slight increase of cytoplasmic FUS (left panel). Cells treated with 0.4 M sorbitol for 1 hr are shown on the right panel. In response to sorbitol stress, slight decrease of nucleus FUS and increase of cytoplasmic FUS-positive inclusion bodies co-localized with eIF4G stress granules were observed. The accumulation of cytoplasmic FUS granules in mutant fibroblasts were much greater than that in healthy controls. Cells were counter stained with the nuclear marker DAPI (blue). Scale bars = 10 μm. (PDF 821 kb) [file 13024_2016_75_MOESM4_ESM.pdf]

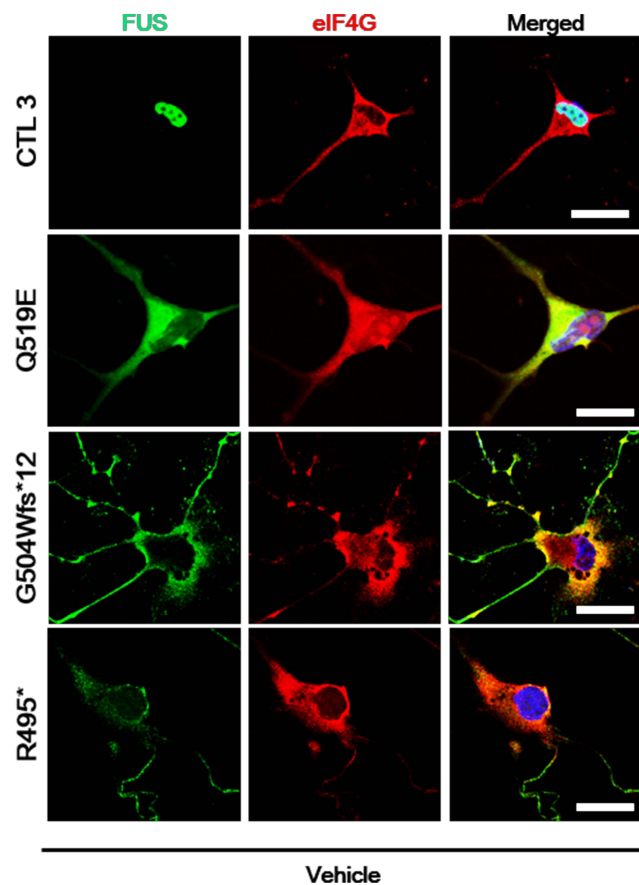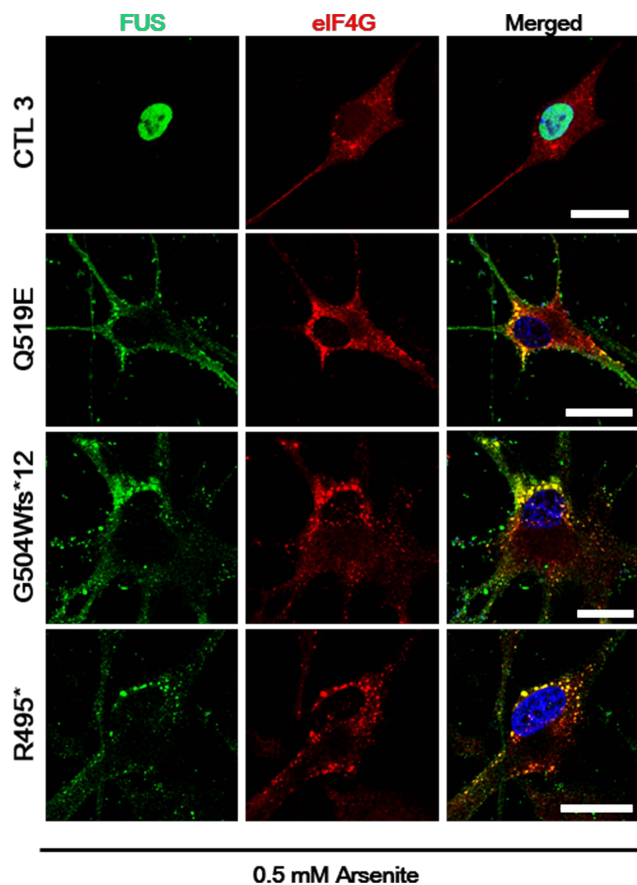

Supplement: Additional file 5: Figure S5. — Endogenous FUS cytoplasmic incorporation into stress granule marker eIF4G in response to arsenite in patient iNeurons. Immunocytochemistry performed on vehicle treated iNeurons (left panel) as compared to cells treated with 0.5 mM arsenite for 30 min (right panel) at day 10 are shown. A representative control shows FUS protein predominantly localized to the nuclei. All three ALS-FUS patients show a majority of FUS protein (green) in the cytoplasm of iNeurons. Cytoplasmic FUS-positive inclusion bodies (green) were detectable in eIF4G-positive stress granules (red) in patients. Cells were fixed and probed by immunofluorescence for DAPI (blue). Scale bars = 25 μm. (PDF 751 kb) [file 13024_2016_75_MOESM5_ESM.pdf]
